# Polish Nursing and Midwifery Master’s Students’ Perceptions of Ethical and Legal Dilemmas Related to Brain Death

**DOI:** 10.3389/ijph.2025.1608625

**Published:** 2025-08-29

**Authors:** Justyna Czekajewska, Dariusz Walkowiak, Anna Jelińska, Jan Domaradzki

**Affiliations:** ^1^ Department of Social Sciences and Humanities, Poznan University of Medical Sciences, Poznań, Poland; ^2^ Department of Organization and Management in Health Care, Poznan University of Medical Sciences, Poznań, Poland; ^3^ Department of Pharmaceutical Chemistry, Poznan University of Medical Sciences, Poznań, Poland; ^4^ Department of Social Sciences and Humanities, Poznan University of Medical Sciences, Poznań, Poland

**Keywords:** ethical and legal dilemmas, nursing and midwifery students, brain death, consent to organ donation, pregnant woman with brain death diagnosis

## Abstract

**Objectives:**

This study analyzes the perceptions of master’s nursing and midwifery students regarding ethical and legal dilemmas related to the declaration of brain death.

**Methods:**

An anonymized, self-administered web-based survey was conducted among 269 master’s students in nursing and midwifery at Poznan University of Medical Sciences.

**Results:**

The most controversial ethical and legal dilemmas concerned the lack of legal consequences for patients’ declarations of will, family objections to organ donation, and sustaining vital functions in pregnant brain-dead patients. While 82.5% accepted the medical definition of brain death, only 53.6% prioritized quality of life over life preservation. Students identified medical knowledge (96.3%) as the most influential factor shaping their attitudes, followed by ethical (66.2%) and religious (45.4%) views. Regression analysis showed that religiosity and age were associated with support for sustaining life functions, while liberal views and a nursing background correlated with greater support for overriding family objections and discontinuing futile therapy.

**Conclusion:**

Education in up-to-date medical knowledge should place greater emphasis on professional ethics, legal frameworks, and real-life bioethical dilemmas to better prepare students for clinical challenges.

## Introduction

A *Definition of Irreversible Coma*, a report by the Harvard Medical School’s Committee to Examine the Definition of Brain Death, played a key role in reformulating the definition of death. It introduced diagnostic criteria for human death, including the lack of perception and reactivity of the nervous system, absence of spontaneous movement for an hour, apnea lasting 3 minutes, lack of reflexes from cranial and peripheral nerves, and an isoelectric EEG line [1]. While the Harvard criteria were not legally binding, *the Uniform Determination of Death Act* (UDDA) was developed in 1981, establishing neurological criteria for death determination [2].

In Poland, brain death–understood as *brain stem death*–refers to the irreversible termination of all cerebral and brain stem functions that cannot resume spontaneously [3]. A new definition was legally regulated by *the Transplantation Act* of 26 October 1995, governing the harvesting and transplantation of cells, tissues, and organs. This law also established clinical guidelines and outlined the specializations of doctors authorized to pronounce death due to neurological causes, based on presumed consent [4]. Initially, instrumental tests were not required.

However, limitations in the Harvard criteria emerged in cases such as extensive facial trauma, drug poisoning, or infratentorial brain injury [5]. Legally, instrumental tests were not required, but in practice, some healthcare facilities used brain blood flow cessation to confirm death, while others rejected this as inconsistent with the law [5]. These clinical, ethical, and legal concerns led to the need for modified legal and diagnostic procedures.

The provisions regarding brain death, previously contained in the Act on the Collection, Storage, and Transplantation of Cells, Tissues, and Organs, have been modificated and incorporated into the Act on the Professions of Physicians and Dentists [6], and the procedure of 4 December 2019, now governs the diagnosis of brain death. It involves repeated clinical assessments, confirmation of brainstem areflexia and permanent apnea, and, when necessary, instrumental tests [7]. Diagnosis must be confirmed by two specialists—one in anesthesiology, intensive care, or neonatology, and the other in neurology, pediatric neurology, or neurosurgery [6, 7].

The introduction of changes to legal regulations on brain death should be accompanied by updates to medical education programs. In accordance with EU directives, a bachelor’s degree in nursing or midwifery is the required qualification for entering professional practice in Poland. Although a master’s degree is not mandatory for licensure, it offers extended clinical, diagnostic, and educational competencies and enables graduates to take on advanced roles such as prescribing medications, issuing referrals, or working in education and leadership positions. It is important to note that midwifery is a distinct and independent field of study, not a specialization within nursing. While both professions share a common foundational framework, midwives follow a separate academic pathway.

Current nursing and midwifery curricula in Poland include subjects such as bioethics, professional ethics, health psychology, neurology, and anesthesiology. These subjects cover various moral and bioethical dilemmas encountered in clinical work, including patient consent and donor care before and after transplantation or in cases of impaired consciousness [8]. However, there is no dedicated module addressing the care of patients diagnosed with brain death, an area that encompasses specific clinical, legal, and ethical challenges for healthcare professionals. Furthermore, practical training related to donor care, communication with families, and discussions about organ donation is also lacking. Therefore, nursing and midwifery education should incorporate a new teaching module focused on brain death. This would better prepare future healthcare providers to navigate the complex realities of patient care in these situations. Without such training, ethical and legal dilemmas are likely to persist.

Research conducted by Majchrowicz has shown that ethical and legal doubts are often present in the work by medical personnel. The researcher found that Polish nurses had an average understanding of brain death criteria, with better knowledge among those aged 41–50 and those frequently assisting in such procedures (64.6%) [5]. Flodén and Forsberg reported that nurses outside intensive care units were more likely to express ethical concerns about brain death [9]. Cohen et al. found that inconsistent definitions of death among healthcare providers negatively impact care for organ donors [10], and promoting organ transplants based on neurological criteria has led to accusations of prioritizing organ procurement over ethical standards [11, 12]. This may shift the medical ethos, allowing one life to be saved at another’s expense [13].

Presumed consent raises further controversy. Without formal consent, the organs of deceased individuals who did not object during life can be used for transplantation, teaching, and research [14]. Difficulties in defining the boundary between life and death also result in concerns over futile therapy–medical actions offering no benefit and often causing suffering by prolonging death and violating dignity [15]. Nonetheless, disputes arise over what constitutes futile treatment. For instance, sustaining the vital functions of a brain-dead pregnant woman might continue solely to support the fetus, even without documented cases of fetal survival or therapeutic benefit for the mother [16, 17].

Accurate diagnosis of consciousness disorders such as minimally conscious state (MCS), vegetative state (VS), coma, and brain death remains scientifically and ethically challenging [18]. Severe brain damage often disrupts both the arousal and awareness components of consciousness [19]. A patient may appear alert without true awareness. Misdiagnosis between MCS and VS is common, despite differing brain mechanisms and prognoses [18]. Improving diagnostic accuracy is essential for rehabilitation and affects how patients are perceived by families and medical professionals.

Recent studies show that an increasing number of brain injury patients, despite appearing unresponsive to stimuli, remain conscious and able to hear their surroundings. They suffer from cognitive-motor dissociation (CMD), a condition that prevents physical responses despite intact cognitive processing. These patients may be mistakenly diagnosed as being in a vegetative state, while in fact they exhibit minimal consciousness [20]. This discovery changes the approach to care-creating an ethical obligation to use diagnostic tools such as fMRI or EEG, and to develop new means of communication (e.g., brain-computer interfaces, implants, or AI-based systems) [21, 22]. In the future, this may influence legal regulations by enabling assessments of patients’ wishes regarding life support, treatment cessation, or organ donation.

Although several studies have shown that nurses and midwives consider some issues related to brain death a source of concern, specific problems have been identified. These include nurses’ fear related to the patient’s death and dying, informing the family of the patient’s death, disconnecting medical equipment supporting a brain-dead patient’s life, and organ donation [5, 23–26]. However, little is currently known about how future nursing and midwifery staff perceive the ethical and legal dilemmas surrounding brain death. This study therefore seeks to analyse the views of master’s students in nursing and midwifery regarding brain death, including 1) ethical and legal dilemmas related to the diagnosis of brain death; 2) factors influencing students’ attitudes towards brain death; and 3) the association between socio-demographic factors and ethical and legal concerns related to the patient’s death by neurologic criteria.

## Methods

### Study Design

The data set includes responses from a self-administered, anonymised pan-and-paper survey on ethical and legal dilemmas related to brain death according to nursing and midwifery students enrolled at the Poznan University of Medical Sciences (PUMS) [27].

### Participants and Setting

Master’s students of nursing and midwifery were targeted for recruitment. The rationale behind choosing these students was two-fold: firstly, after completing the first stage of studies (3 years), which concludes with a bachelor’s degree in nursing or midwifery respondents are already qualified healthcare professionals and, during the second stage (2 years), i.e., their master’s studies, most students are already engaged professionally in a variety of healthcare facilities, and secondly, they were liable to face ethical and legal dilemmas related to death and dying patients.

The inclusion criteria were as follows: 1) being a master’s student of nursing or midwifery, 2) being enrolled at PUMS, 3) speaking Polish, 4) being willing to participate in the study, and 4) providing written informed consent before completing the survey.

### Research Tools

The questionnaire used in this study was constructed once published literature on the attitudes of healthcare professionals towards ethical and legal dilemmas surrounding the definition and diagnosis of brain death had been reviewed [26, 28–32]. It was developed in line with the guidelines of the European Statistical System [33]. A group of experts: a medical sociologist, a bioethicist and public health specialists, was consulted regarding the first draft of the questionnaire. It was then pre-tested in a pilot study with a group of 115 bachelor’s students of midwifery and medical rescue, which resulted in the reformulation of three questions.

The final version of the questionnaire consisted of three parts. The first asked questions on the midwifery master’s and nursing students’ opinions on their ethical and legal dilemmas related to the diagnosis of brain death. The second part comprised questions about factors shaping respondents’ attitudes towards death. The last part of the questionnaire included questions about the students’ demographic characteristics.

All items were designed as close-ended questions with a limited set of pre-defined and simple answers to choose from. Questions were explored using closed-ended questions using a 5-Likert scale ranging from 1 (strong dissatisfaction or disagreement) to 5 (high satisfaction or agreement). They also contained the neutral answer *I do not know*. The survey questions were moreover expressed in plain language with no technical jargon.

### Data Collection

The study was conducted between March and June 2024 among master’s students of nursing and midwifery at PUMS. Convenience sampling was used in this survey. Participants received neither monetary nor non-monetary compensation for completing the questionnaire.

The student participants were recruited during regular classes. Before completing the survey, all students were informed by the principal investigator (JC) about the study’s aim, as well as its voluntary, anonymous, confidential and non-compensatory character. Participants were also instructed about their right to withdraw from the survey at any given moment without any consequences. Following an explanation of the study’s goal, participants were given questionnaires at seminars and had an opportunity to ask questions during the data collection procedure. After informed written consent was obtained from all students who were willing to participate in the survey, all respondents competed the questionnaire with pen and paper. Completing the questionnaire took between 10 and 15 min.

### Ethical Issues

This study was conducted in accordance with the principles set forth in the Declaration of Helsinki [34]. Prior to the commencement of the study, ethical and research governance approval was obtained from the Poznan University of Medical Sciences Bioethics Committee (KB – 07/24, granted on 3rd January 2024).

### Data Analysis

Descriptive statistics were used to summarize sociodemographic characteristics and responses to Likert-scale questions, reported as frequencies and percentages of the total sample. To assess the influence of sociodemographic variables on attitudes toward ethical and legal dilemmas related to brain death, stepwise logistic regression was conducted using JASP (Version 0.18.3). The dependent variable captured respondents’ ethical stances on brain death and its diagnostic criteria, while the independent variables comprised demographic characteristics and other relevant factors listed in Table 1. A stepwise selection method was used to iteratively include variables that made a statistically significant contribution to the model. To enhance readability and reduce redundancy, both odds ratios and 95% confidence intervals are reported for all predictors. A p-value of <0.05 was considered statistically significant [35].

**TABLE 1:** Socio-demographic characteristics of the study participants (Poland, 2024).

Characteristics	Total	
	(n = 269)	%
Gender		
Female	262	97.4
Male	5	1.9
Non-Binary	2	0.7
Faculty		
Nursing	125	46.5
Midwifery	144	53.5
Year of the study		
4	123	45.7
5	146	54.3
Age (in years)		
Range	22–50	
Mean (95%CI)	23.9 (23.5–24.2)	
SD	3.1	
Median	23	
Professional internship		
Yes	227	84.4
No	42	15.6
Role of religion plays in your life		
Significant/rather significant	79	29.4
None/little	190	70.6
Liberal–conservative orientation (worldview beliefs)		
Definitely liberal/rather liberal	149	55.4
Centre	102	37.9
Rather conservative/definitely conservative	18	6.7

## Results

Out of 293 students approached, 269 participated (response rate: 91.8%) (Table 1). The sample was predominantly female (97.4%), reflecting gender patterns in Polish medical professions, particularly in midwifery (99.8%) and nursing (96.4%). Participants were nearly evenly split between nursing (46.5%) and midwifery (53.5%) students, mostly in their fourth (45.7%) or fifth (54.3%) year of study. Ages ranged from 22 to 50 (mean: 23.9, SD: 3.1). Most had completed internships (84.4%). Religion was of little importance to 70.6%, and over half identified as liberal (55.4%), with 37.9% centrist and 6.7% conservative.

Table 2 summarizes responses to ethical questions about brain death. Most (82.5%) accepted the medical definition of brain death; 72.9% opposed maintaining life support in such cases. Views were mixed on disconnecting support despite family objections (45.4% opposed, 34.9% supported). While 76.6% supported organ donation based on declared consent, 52.8% opposed overriding family wishes. A majority supported sustaining life functions in pregnant brain-dead patients (65.1%) and rejected equating vegetative states with death (87.4%). Opinions were divided on religious input in determining brain death (46.1% for, 43.5% against). Just over half (53.6%) prioritized quality of life over life preservation.

**TABLE 2:** Ethical dilemmas of respondents regarding brain death and the criteria for its diagnosis (Poland, 2024).

Item	Definitely not n (%)	Rather not n (%)	I do not know n (%)	Rather yes n (%)	Definitely yes n (%)
The concept of medical brain death is understood as permanent loss of consciousness and permanent loss of all brainstem functions	4 (1.5)	10 (3.7)	33 (12.3)	137 (50.9)	85 (31.6)
In the case of a diagnosis of brain death should life-sustaining functions be maintained in the patient	67 (24.9)	129 (48)	35 (13)	31 (11.5)	7 (2.6)
A doctor may order the disconnection of a patient’s life support equipment despite the family’s objection	44 (16.4)	78 (29)	53 (19.7)	80 (29.7)	14 (5.2)
When brain death is diagnosed in a patient who has made a declaration of will, consent to organ donation still needs to be given	16 (5.9)	15 (5.6)	32 (11.9)	103 (38.3)	103 (38.3)
If a patient diagnosed with brain death has not objected to organ donation, the doctors should be able to make an independent decision against the family’s wishes if they object	71 (26.4)	71 (26.4)	23 (8.6)	66 (24.5)	38 (14.1)
A brain death diagnosis in a pregnant patient obligates the medical staff to sustain her life functions to maintain the pregnancy	8 (3)	19 (7.1)	67 (24.9)	114 (42.4)	61 (22.7)
The court should grant permission to disconnect life-support equipment in the case of the diagnosis of brain death in a child despite the family’s possible objection	30 (11.2)	65 (24.2)	57 (21.2)	86 (32)	31 (11.5)
A patient’s religious beliefs regarding life and death should be considered when making decisions about determining brain death	47 (17.5)	70 (26)	28 (10.4)	90 (33.5)	34 (12.6)
State of deep consciousness disorder (coma) is a reason to consider a patient deceased	139 (51.7)	96 (35.7)	24 (8.9)	8 (3)	2 (0.7)
A patient in a vegetative state (for at least 5 weeks) after being diagnosed with a coma should be considered deceased	105 (39)	99 (36.8)	47 (17.5)	17 (6.3)	1 (0.4)
A patient in a persistent vegetative state (3–6 months after being diagnosed with coma) should be considered deceased	86 (32)	91 (33.8)	60 (22.3)	30 (11.2)	2 (0.7)
A patient who shows a minimal level of consciousness may be considered deceased	55 (20.4)	121 (45)	57 (21.2)	34 (12.6)	2 (0.7)
Futile therapy should be discontinued in patients diagnosed with brain death	13 (4.8)	20 (7.4)	55 (20.4)	106 (39.4)	75 (27.9)
Quality of life is more important than life itself	21 (7.8)	49 (18.2)	55 (20.4)	90 (33.5)	54 (20.1)
Saving the life of a patient at the cost of the life of another person, who has no chance of survival without medical equipment, is an ethically permissible act	27 (10)	53 (19.7)	83 (30.9)	80 (29.7)	26 (9.7)
New regulations on brain death have influenced a change in society’s views on human death, prioritising neurological criteria	6 (2.2)	72 (26.8)	116 (43.1)	67 (24.9)	8 (3)

Table 3 shows students’ subjective evaluations of the importance assigned to factors influencing attitudes toward death: medical knowledge (96.3%) was considered most significant, followed by ethical/moral views (66.2%) and legal norms (65.5%). Religious beliefs were important to 39.1% but irrelevant to 45.4%, while philosophical, cultural, and political views were less influential.

**TABLE 3:** Students’ perceptions of factors influencing their attitudes toward brain death (Poland, 2024).

What is central to your understanding of what death is?	Definitely not n (%)	Rather not n (%)	I do not know n (%)	Rather yes n (%)	Definitely yes n (%)
Medical knowledge	4 (1.5)	5 (1.9)	1 (0.4)	38 (14.1)	221 (82.2)
Religious beliefs	78 (29)	44 (16.4)	42 (15.6)	68 (25.3)	37 (13.8)
Ethical/moral views	17 (6.3)	42 (15.6)	32 (11.9)	125 (46.5)	53 (19.7)
Philosophical views	51 (19)	79 (29.4)	69 (25.7)	61 (22.7)	9 (3.3)
Legal norms	24 (8.9)	39 (14.5)	30 (11.2)	111 (41.3)	65 (24.2)
Cultural norms	46 (17.1)	65 (24.2)	61 (22.7)	79 (29.4)	18 (6.7)
Political views	127 (47.2)	92 (34.2)	29 (10.8)	17 (6.3)	4 (1.5)

Regression analysis (Table 4) identified several predictors. Religious and older students were more likely to support maintaining life support (OR = 2.086 and 1.143, respectively). For organ donation with declared consent, religious (OR = 3.928), liberal (OR = 8.214), and midwifery students (OR = 1.990) were more supportive. In contrast, religious and conservative respondents, as well as midwifery students, were less likely to support overriding family objections. Discontinuation of futile therapy was more supported by liberals (OR = 2.843), but less by older individuals and midwifery students. The model explaining attitudes toward futile therapy had the highest explanatory power (R^2^ = 0.201).

**TABLE 4:** Regression analysis results (Poland, 2024).

Regression parameters	1. The concept of medical brain death is understood as permanent loss of consciousness and permanent loss of all brainstem functions	2. In the case of a diagnosis of brain death should life-sustaining functions be maintained in the patient	3. When brain death is diagnosed in a patient who has made a declaration of will, consent to organ donation still needs to be given	4. If a patient diagnosed with brain death has not objected to organ donation, the doctors should be able to make an independent decision against the family’s wishes if they object	5. A brain death diagnosis in a pregnant patient obligates the medical staff to sustain her life functions to maintain the pregnancy	6. Futile therapy should be discontinued in patients diagnosed with brain death
	OR (95% CI)	OR (95% CI)	OR (95% CI)	OR (95% CI)	OR (95% CI)	OR (95% CI)
Intercept		0.005*** (0.000–0.057)	0.370 (0.096–1.419)	3.803* (1.132–12.770)		216.383** (4.195–11160.858)
Age		1.143** (1.036–1.256)				0.825* (0.701–0.971)
Midwifery vs. Nursing			1.990** (1.096–3.613)	0.340*** (0.200–0.576)		0.255*** (0.143–0.457)
Religious vs. Nonreligious		2.086* (1.010–4.309)	3.928** (1.644–9.386)	0.491* (0.242–0.994)		
Liberal vs. Conservative			8.214** (2.147–31.430)			2.843* (1.001–8.310)
Conservative vs. Centre				0.192** (0.061–0.607)		
R2 Nagelkerke	0	0.086	0.124	0.160	0	0.201
p-value for Model	0	0.05	<0.001	0.043	0	0.042

Notes: * p-value <0.05; ** p-value <0.01; *** p-value <0.001.

## Discussion

The results of this study show that although most nursing and midwifery students correctly understand the medical definition of brain death as the permanent loss of consciousness and all brain stem functions, they identified several ethical and legal dilemmas in caring for brain-dead patients. Concerns are particularly focused on the legal principles of organ harvesting from deceased donors and maintaining the vital functions of brain-dead pregnant patients.

Previous studies show varying levels of knowledge among healthcare professionals regarding brain death declaration. Majchrowicz reported that 94.2% of Polish nurses understood the medical definition of brain death, and 93.7% supported life support cessation after clinical diagnosis. Respondents with experience assisting in brain death diagnosis answered more accurately [5]. Similarly, 70% of Spanish nurses knew the criteria, with final-year students scoring higher than first-year students (81% vs. 59%) [36]. In contrast, studies in Turkey found that most medical students were unaware of diagnostic criteria, and nurses confused coma with brain death [37, 38]. Tawil et al. attributed students’ knowledge gaps to insufficient training on neurological causes of death [39]. These findings suggest knowledge depends on both experience with neurological death cases and relevant educational courses.

However, medical understanding does not always align with legal knowledge. In this study, 52.8% disagreed with organ donation if the family objected, while 76.6% supported transplantation if the deceased signed a declaration of will. This suggests a poor understanding of Poland’s legal framework on organ procurement. Michalska et al. found that although 91% of paramedic students had heard of the declaration of will and 63% of the Central Register of Objections, only 36% answered correctly regarding the need to verify objections before harvesting organs. Half were unaware of the objection process, and 72.7% mistakenly thought families could object [40]. Similarly, Hryciuk et al. found most Polish respondents knew of the declaration of will (70.9%) but lacked legal knowledge only 33% answered relevant questions correctly [41]. Czaplińska et al. confirmed this, with just 19.3% knowing that a signature alone does not authorize organ harvesting [42]. These results indicate that, although students and medical staff have a good understanding of the definition of brain death, they are unaware of its legal implications. The difference between the medical definition of brain death and the knowledge of the legal status of transplantation and presumed consent stems primarily from the distinct nature of these issues. Brain death means the irreversible cessation of all brain functions, which legally equates to the patient’s death [3]. This definition is based on neurological criteria and is fundamental to the decision to terminate life-sustaining treatment and initiate organ harvesting procedures for transplantation. Presumed consent, in turn, is legally regulated by the Act on the Collection, Storage, and Transplantation of Cells, Tissues, and Organs. In Poland, the principle of presumed consent applies–this means that a citizen who has not expressed an objection during their lifetime (e.g., by entering it in the Central Register of Objections) can be a potential donor after brain death has been confirmed [4]. The lack of fundamental understanding of these issues may result from differences in curricula between fields of study, shortcomings in pre- and postgraduate education, and a lack of standardization in the teaching of bioethics and medical law. Although nursing and midwifery curricula include courses in bioethics, professional ethics, neurology, and psychology, topics related to brain death, transplantology, and medical law are discussed superficially and theoretically, without any reference to the integration of clinical knowledge with law and ethics [8]. Unlike in the medical field, in midwifery and nursing, knowledge of these topics is limited to a few subjects and is not developed in depth [8]. Curricula also vary significantly between universities, resulting in a lack of central guidelines regarding the scope of discussion of issues related to brain death and transplantology.

Furthermore, misunderstandings of legal provisions may also lead to the rejection of the presumed consent model. More than half of the respondents in many Western European countries prefer the expressed consent model. In the Netherlands, 40% expressed support for consent vs. 25% for presumed; in Ireland, 62% support maintaining expressed consent; similarly in Germany (62% vs. 38%). In contrast, Iceland (80.4% vs. 11.9%) and Scotland (nearly 55%) showed stronger support for presumed consent [43]. Among Polish students, medical students had the highest awareness (86.9% vs. 42.6%) and greater understanding of presumed consent (62% vs. 23.5%) [44]. Despite high social acceptance of transplantology (80%), 46% declared lacking knowledge of legal regulations, and 29% answered incorrectly [45]. Western Europe remains divided between the two models: presumed consent increases organ availability and recipient survival, while expressed consent emphasizes autonomy but requires costly public education efforts.

The lack of full acceptance of the presumed consent model may also stem from deeply rooted family values and a willing to avoid conflict between medical staff and the patient’s family. In Polish society, there is a prevailing belief that the deceased’s next of kin should have the decisive voice in matters concerning the body after death, regardless of the legal framework in place. A nationwide study in Poland found that a significant portion of respondents (ranging from 42.6% to 64.6%, depending on educational level) believed that the decision to retrieve organs should depend primarily on the family’s consent, rather than legal regulations alone [46]. In countries such as Poland and Spain, despite the legal implementation of a presumed consent system (soft opt-out), consultations with the deceased’s family are a standard clinical practice [47, 48]. Although the family does not formally hold veto power, informing them of the planned organ retrieval- and in many cases, obtaining their approval - is considered not only ethically appropriate but also pragmatically necessary. Medical personnel engage in discussions with the family out of respect for their emotions, but also due to concerns about potential legal, social, and emotional repercussions. The fear of being accused of a lack of empathy, triggering interpersonal conflict, or even facing legal action, often compels healthcare providers to include the family in the decision-making process. Omitting such consultation could lead to serious consequences - not only in terms of the relationship with the deceased’s relatives but also for the overall transplant system. A lack of transparency and openness with the patient’s family could erode public trust in medical professionals and diminish broader societal support for organ donation. Therefore, although the law permits organ retrieval without family consent, in practice, the role of the next of kin is not questioned - in fact, their acceptance is often treated as a condition for the social legitimacy of the entire transplantation process.

Pregnant patients in ICUs present another complex ethical and legal dilemma. Some scholars argue this issue remains unresolved due to unclear clinical and ethical guidelines for pregnant women with severe brain injuries [49]. Polish law deems therapy futile and unethical once brain death is diagnosed [15, 50]. According to 69% of medical students, futile therapy is discontinued in fewer than 30% of cases [51]. In one study, 66% of nurses from ICUs and other units reported participating in futile therapy [52], with about 55% citing fear of legal consequences and 46% citing fear of family reactions as key reasons [53]. While most respondents favor ending futile therapy [54, 55], they believe the decision should be based on prognosis and patient wishes [54].

Two exceptions to this are maintaining organ function for donation and supporting vital functions of brain-dead pregnant women. Pikto-Pietkiewicz et al. noted the latter scenario is rare and poorly defined [56]. As fetal life depends on the mother’s biological function, prolonging maternal life support may be justified [57, 58]. Before doing so, researchers recommend considering gestational age [16], the effect of drugs and diagnostics on the fetus [49, 59], and providing multidisciplinary care to improve fetal outcomes [49, 59]. Ethical and legal issues arise from maintaining a deceased woman’s body for gestation and making decisions on her behalf. In the absence of clear directives, physicians often assume that a woman who did not plan to terminate the pregnancy would want the child to be born [58].

Medical knowledge plays a critical role in shaping attitudes toward brain death. In this study, 96.3% of respondents agreed it influenced their perspective. Because nursing and midwifery involve direct patient care, academic and clinical training are key. Some studies show experience and years of study correlate with better knowledge [60, 61]. Another study found students preferred specialist lectures over the formal curriculum (55.3% vs. 15.8%) [62].

Socio-demographic factors, including field of study and religiosity, also shape ethical and legal views. Studies show religious beliefs influence views on death and organ donation. Scientific definitions of brain death are more accepted by Jews (71%), Protestants (52%), and non-believers (59%) than by Buddhists (83%), Hindus (72%), and Muslims (62%), who often oppose neurological death criteria. Regarding legal organ donation, Jews, non-believers (69%), Catholics, and Protestants (61%) were more supportive than Buddhists, Hindus, and Muslims (31%) [63]. Alhawari et al. found religion strongly influences views on death and transplantology, aligning closely with the norms of each faith [64, 65]. Skepticism about brain death may stem from its diagnostic uncertainty [63]. Cebulska, Koźlak, and Dybalski found religiosity had more influence on attitudes toward futile therapy than age, experience, education, or residence. Among Catholics, 97% supported protocols for withdrawing futile treatment when no benefit exists [54].

The analysis of the conducted logistic regression revealed that students who declare themselves as religious and hold conservative political views are more likely to experience ethical dilemmas. This is reflected in their greater tendency to support the continuation of life-sustaining treatment and their preference for decisions regarding postmortem organ donation to be made in consultation with the deceased patient’s family - even when the prevailing legal framework allows for a presumed consent model. These results may indicate a deeper adherence to values rooted in personalist ethics, the inviolability of the body after death, the strong role of the family, loyalty to the community, and skepticism toward state intervention in bodily and postmortem decisions [66–68]. A different approach was observed among students with liberal and non-religious views, who more frequently accept the termination of futile therapy and support organ donation following brain death. Their attitudes can be interpreted through the lens of utilitarian beliefs, based on the idea of maximizing benefits and minimizing suffering. The willingness to discontinue ineffective treatment - which offers no hope of recovery and prolongs suffering - as well as a positive attitude toward transplantation as a form of helping others, suggests that they perceive donation not as a violation of bodily sanctity, but rather as a continuation of doing good after death [69, 70].

An exception in ethical attitudes concerns cases of pregnant women diagnosed with brain death. As shown by Alhawari et al., even when brain death was recognized as irreversible, the majority of respondents from major religious traditions - Catholics (59%), Protestants (55%), and Buddhists (70%) - still perceived such patients as alive. This further highlights the complexity of beliefs surrounding the beginning and end of life [63].

This study also found diverging views. While 82.5% accepted the medical definition of death, 65.1% supported maintaining vital functions in brain-dead pregnant patients. These views may stem from doubts about the irreversibility of brain death or from adherence to professional ethics. When facing ethical dilemmas, nurses and midwives prioritize patient and family wellbeing. Decision-making can be complex due to competing interests and values. Some studies show that caring for terminal patients heightens moral sensitivity [71–73]. To address this, researchers recommend enhancing ethical education in nursing programs [65].

Research also indicates that nurses are less satisfied than doctors with end-of-life care, more likely to witness moral issues, and more likely to consider leaving their job due to ethical concerns. Improving the ethical climate in ICUs through open dialogue and better cooperation between nurses and doctors is advised [73].

### Limitations

Although this study has shed some light on the ethical and legal dilemmas regarding brain death faced by master’s students of nursing and midwifery, it has some limitations. Firstly, since the study involved nursing and midwifery students from only one Polish medical university, it has a local dimension, and the results cannot be extrapolated to represent all nursing and midwifery students, either in Poznan or across Poland. Consequently, it would be desirable to compare the findings with data from other locations in the country. Secondly, the future studies should aim for more representative methods, such as random or stratified sampling, to better capture the heterogeneity of this population. Thirdly, female students outnumbered male participants. However, it should be noted that all medical studies in Poland, and especially nursing and midwifery courses, are strongly gendered. The gender imbalance results from structural and cultural patterns in Polish medical education. In 2021, women made up 74% of all students in medical and healthcare programs nationwide, with this share rising to 89% among nursing students and 99% among midwifery students. At PUMS alone, women accounted for 76% of all students enrolled in medical and health-related programs in 2023 [74, 75]. These data underscore the extent to which both fields remain heavily feminized in Poland, shaped largely by persistent societal perceptions and occupational stereotypes regarding gender roles in healthcare. Fourthly, future studies might also include other students who have contact with dying patients, i.e., medicine, psychology and emergency medicine, as caring for brain-dead patients also concerns students of these disciplines, who in clinical practice work as part of interdisciplinary teams. Fifthly, as this study is based solely on a quantitative method, further in-depth research using qualitative methods would be necessary to better understand the ethical and legal concerns of nurses and midwives, especially in terms of the personal, emotional, and cultural dimensions that cannot be fully captured through standardized questionnaires, and to explore how these concerns manifest in real clinical settings and decision-making processes. Finally, since our analysis focused on students’ opinions on ethical and legal dilemmas related to brain death rather than actual dilemmas and decisions faced during clinical practice, it is important to note that stated intentions do not always align with real-life behavior.

### Conclusion

Although most students declared that they accepted the medical definition of brain death, many of them experienced ethical and legal dilemmas related to providing care to a brain-dead patient. Some important implications therefore emerged from this study:

1. Students of nursing and midwifery should be better trained in palliative care competencies. This could be achieved through the inclusion of additional mandatory modules on palliative and end-of-life care in master’s degree curricula, coordinated by medical universities and supported by national nursing chambers. In addition to existing clinical placements, promoting student volunteering in hospices and palliative care wards could offer valuable first-hand experience, foster empathy, and enhance practical competencies in caring for dying patients.
2. Although students declared that medical knowledge had the greatest influence on their attitudes toward brain death, they show deficits in the ability to solve ethical and legal dilemmas related to caring for a brain-dead patient. Medical education should, therefore, emphasize education in the field of bioethics and medical law. These subjects should be taught in both theoretical and case-based formats, and integrated into existing clinical training. Curriculum development could be overseen by university program councils in consultation with professional organizations.
3. Students should be taught bioethical dilemmas by describing real case studies related to the professional experience of nurses and midwives. Simulation-based learning, such as role-playing, standardized patient scenarios, or high-fidelity clinical simulations, can provide a safe and structured environment to explore ethically complex situations and practice communication and decision-making skills. Seminars led by experienced practitioners could further enhance students’ moral reasoning and critical thinking. This approach could be implemented jointly by academic institutions and continuing education providers.
4. Medical education should also include information about death from various religious and philosophical traditions in order to deepen students’ knowledge and better respond to the needs of patients with different worldviews and religious beliefs. Optional interdisciplinary courses or guest lectures involving experts in medical humanities, theology, or intercultural communication could be offered at medical universities and supported through collaboration with ethics committees or professional nursing associations.

## Data Availability

The data supporting this study’s findings are not openly available due to reasons of sensitivity and are available from the corresponding author upon reasonable request. Data are located in controlled access data storage at Poznan University of Medical Sciences.

## Abbreviations

EEG, electroencephalography; UDDA, the Uniform Determination of Death Act; MCS, minimally conscious state; VS, vegetative state; PUMS, Poznan University of Medical Sciences; PSV, persistent vegetative state.
